# The TRPC1 Channel Forms a PI3K/CaM Complex and Regulates Pancreatic Ductal Adenocarcinoma Cell Proliferation in a Ca^2+^-Independent Manner

**DOI:** 10.3390/ijms23147923

**Published:** 2022-07-18

**Authors:** Julie Schnipper, Sana Kouba, Frédéric Hague, Alban Girault, Pierre Rybarczyk, Marie-Sophie Telliez, Stéphanie Guénin, Riad Tebbakha, Henri Sevestre, Ahmed Ahidouch, Stine Falsig Pedersen, Halima Ouadid-Ahidouch

**Affiliations:** 1 Laboratory of Cellular and Molecular Physiology, UR UPJV 4667, University of Picardie Jules Verne, 80000 Amiens, France; julie_schnipper@hotmail.com (J.S.); sana.kouba@hotmail.com (S.K.); fh-lnc@u-picardie.fr (F.H.); alban.girault@u-picardie.fr (A.G.); rybarczyk.pierre@chu-amiens.fr (P.R.); marie-sophie.telliez@u-picardie.fr (M.-S.T.); tebbakha.riad@chu-amiens.fr (R.T.); henrisevestre@gmail.com (H.S.); ahidouch@gmail.com (A.A.); 2 Anatomy and Pathology Department, Amiens Hospital, University of Picardie Jules Verne, Tumorotheque of Picardie, 80000 Amiens, France; 3 Centre de Ressources Régional en Biologie Moléculaire, Université de Picardie Jules Verne, 80000 Amiens, France; stephanie.vandecasteele@u-picardie.fr; 4 Biology Department, University Ibn Zohr, Agadir 80000, Morocco; 5 Section for Cell Biology and Physiology, Department of Biology, University of Copenhagen, 1165 Copenhagen Ø, Denmark; sfpedersen@bio.ku.dk

**Keywords:** pancreatic ductal adenocarcinoma, TRPC1, cell proliferation, spheroid growth, cell cycle progression, PI3K, calmodulin

## Abstract

Dysregulation of the transient receptor canonical ion channel (TRPC1) has been found in several cancer types, yet the underlying molecular mechanisms through which TRPC1 impacts pancreatic ductal adenocarcinoma (PDAC) cell proliferation are incompletely understood. Here, we found that TRPC1 is upregulated in human PDAC tissue compared to adjacent pancreatic tissue and this higher expression correlates with low overall survival. TRPC1 is, as well, upregulated in the aggressive PDAC cell line PANC-1, compared to a duct-like cell line, and its knockdown (KD) reduced cell proliferation along with PANC-1 3D spheroid growth by arresting cells in the G1/S phase whilst decreasing cyclin A, CDK2, CDK6, and increasing p21^CIP1^ expression. In addition, the KD of TRPC1 neither affected Ca^2+^ influx nor store-operated Ca^2+^ entry (SOCE) and reduced cell proliferation independently of extracellular calcium. Interestingly, TRPC1 interacted with the PI3K-p85α subunit and calmodulin (CaM); both the CaM protein level and AKT phosphorylation were reduced upon TRPC1 KD. In conclusion, our results show that TRPC1 regulates PDAC cell proliferation and cell cycle progression by interacting with PI3K-p85α and CaM through a Ca^2+^-independent pathway.

## 1. Introduction

Pancreatic ductal adenocarcinoma (PDAC) is the most prevalent type of pancreatic cancer, accounting for more than 90% of all pancreatic malignancies [1]. With a mortality rate similar to its incidence, the 5-year survival rate remains one of the lowest (less than 10%) of all cancers [2]. When detected, PDAC is often at a late disease stage. Overall, the response rate for late-diagnosed patients is limited. The poor prognosis of PDAC patients is associated with rapid tumor progression and limited available treatments [3].

Calcium (Ca^2+^) is a ubiquitous intracellular messenger that transduces signals in cellular processes, including cell fertilization, differentiation, cell growth, and death [4]. The influx of Ca^2+^ through Ca^2+^ channels plays an important role in cell cycle progression in several cell types [5,6]. However, the direct relationship between Ca^2+^ and cellular proliferation is complex, as many cell types can proliferate even in the near absence of extracellular Ca^2+^ [7]. The transient receptor potential (TRP) and store-operated channels (SOC) are recognized as major regulators of the Ca^2+^ influx in epithelial cancer cells [8]. Growing evidence demonstrates that the TRP canonical (TRPC) channel subfamily is involved in tumor development [9,10]. The first identified member of this non-selective cation channel subfamily is TRPC1. TRPC1 is a potential regulator of store-operated Ca^2+^ entry (SOCE) pathways [11] and has shown to be a potential biomarker of different cancer types, as its dysregulation correlates with several clinical parameters, including overall survival [12,13,14,15,16,17,18,19,20,21]. At the cellular level, TRPC1 contributes to a plethora of physiological and pathophysiological processes, including tumor cell proliferation and growth. However, it seems that it depends on the cancer cell type, and whether the presence or absence of TRPC1 contributes to cell growth. In a majority of studies, TRPC1 downregulation inhibits the proliferative rate in neuroblastoma [22], glioblastoma [23], thyroid [24], breast [12,25,26,27], lung, [28], liver [29,30], ovarian [31], and colon cancer [13]. Whereas in other studies, TRPC1 downregulation drives proliferation and growth in esophageal [32] and breast cancer [33]. Additionally, it is controversial whether the influx of Ca^2+^ causes this proliferation through TRPC1. In different cancer cell models, the depletion of TRPC1 diminishes SOCE [12,23,27,28,29], whereas, in others, this increased or did not affect SOCE [13,24,30,34,35,36,37]. TRPC1 principally regulates proliferation through the phosphoinositol-3-kinase (PI3K) and mitogen-activated protein kinase (MAPK) pathways in several cancers [13,26,28,38]. TRPC1 regulates PI3K and its downstream signaling kinases, such as AKT, differently, depending on whether it is an activator or an inhibitor of proliferation. Thus, in the lung (A549) and hepatocellular (Huh7) cancer cell lines, the depletion of TRPC1 inhibits cell proliferation by decreasing the activation of AKT [28,29]. Meanwhile, TRPC1 downregulation enhances cell proliferation by activating AKT in the esophageal (EC9706 cells) and breast (MCF-7 cells) carcinoma cell lines [32]. Recently, it has been shown that TRPC1 interacts with PI3K through calmodulin (CaM). Here, CaM, a multifunctional intracellular Ca^2+^-binding protein, works as a connecting protein between TRPC1 and PI3K, thereby regulating colorectal cancer progression [13].

Nonetheless, to the best of our knowledge, nothing is known about TRPC1 expression in PDAC tumors and the possible downstream mechanisms that contribute to PDAC cell proliferation. In the present study, we demonstrated that TRPC1 upregulation correlates with low overall survival in PDAC patients. Furthermore, we revealed that TRPC1 regulates cell proliferation and cell cycle progression, particularly in aggressive PDAC cells, independently from Ca^2+^-entry, by forming a complex with the PI3K p85α subunit and CaM, leading to activation of AKT.

## 2. Results

### 2.1. TRPC1 Upregulation Correlates with the Aggressive Basal-like Subtype of PDAC and with Low Overall Survival

**Figure 1:**
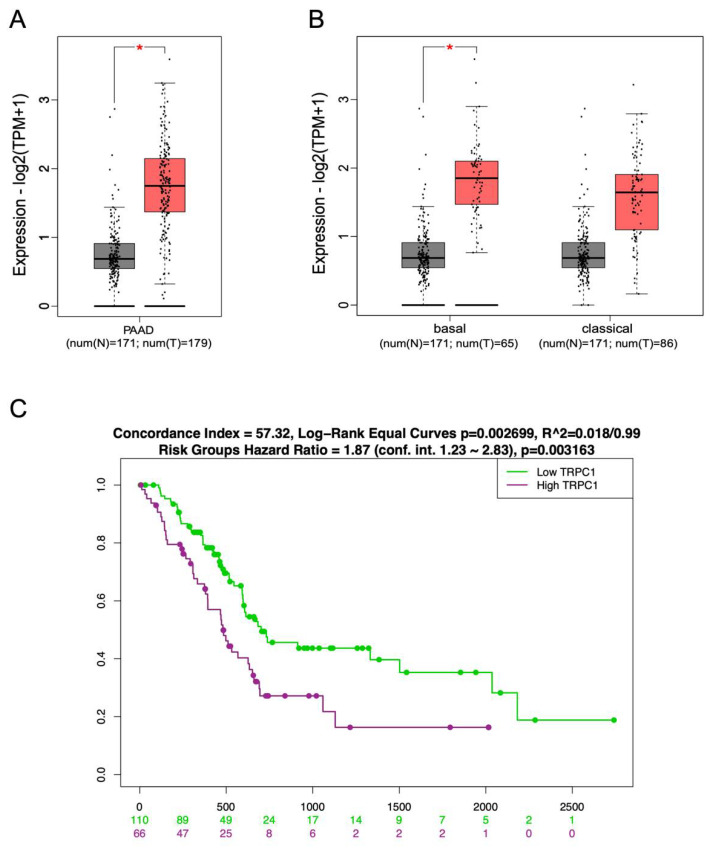
TRPC1 overexpression correlates with poor overall survival in PDAC patients. (**A**) Whisker boxplots of TRPC1 mRNA expression in normal samples (N) and PDAC tumor tissues (T) were generated by GEPIA2 using data from The Cancer Genome Atlas (TCGA) cohort PAAD and Genotype-Tissue Expression (GTEx) samples. * Indicates *p* < 0.01. (**B**) Whisker boxplots of TRPC1 mRNA expression in normal samples (N) and PDAC tumor tissues (T) in both the basal and classical subtypes of PDAC were generated by GEPIA2 using data from the TCGA cohort PAAD and GTEx samples. * Indicates *p* < 0.01. (**C**) Overall survival analysis of patients within the PAAD cohort with either a high or low expression of TRPC1 was generated using SurvExpress. The Kaplan–Meier curve was analyzed using the optimized SurvExpress Maximize algorithm. The number of analyzed patients across time (days) is indicated below the horizontal axis.

The expression of TRPC1 has been shown to be dysregulated in several types of cancer [1,2,3,4] and was recently shown to be correlated with poor prognosis in colorectal cancer [5]. However, little is known about its expression in PDAC. Therefore, we analyzed the expression of TRPC1 in normal pancreatic tissue and human PDAC samples using the PAAD TCGA dataset of normal and tumor samples. The results showed that TRPC1 mRNA expression was significantly upregulated in PDAC tissue samples compared to non-tumor tissue samples (N = 171 vs. 179, *p* < 0.01, Figure 1A). Furthermore, the results showed that the upregulation of TRPC1 was significantly higher in samples of a more aggressive basal-like PDAC subtype (N = 171 vs. 65 *p* < 0.01, Figure 1B) compared to TRPC1 expression in the classical PDAC subtype (N = 171 vs. 86, Figure 1B). To investigate the prognostic significance of TRPC1, we analyzed whether TRPC1 overexpression was associated with survival in PDAC patients. The Kaplan–Meier survival analysis using SurvExpress, an online resource to correlate gene expression with overall survival, showed that patients with high levels of TRPC1 (N = 110) expression were associated with a significantly low overall survival compared to patients with low expression (N = 66, *p* < 0.01, Figure 1C). Altogether, these online available expression data reveal that TRPC1 is overexpressed in human PDAC tissue, which correlates with low overall survival.

### 2.2. TRPC1 Is Overexpressed in Cell Lines with an Aggressive Phenotype and Is Localized to the Plasma Membrane

**Figure 2:**
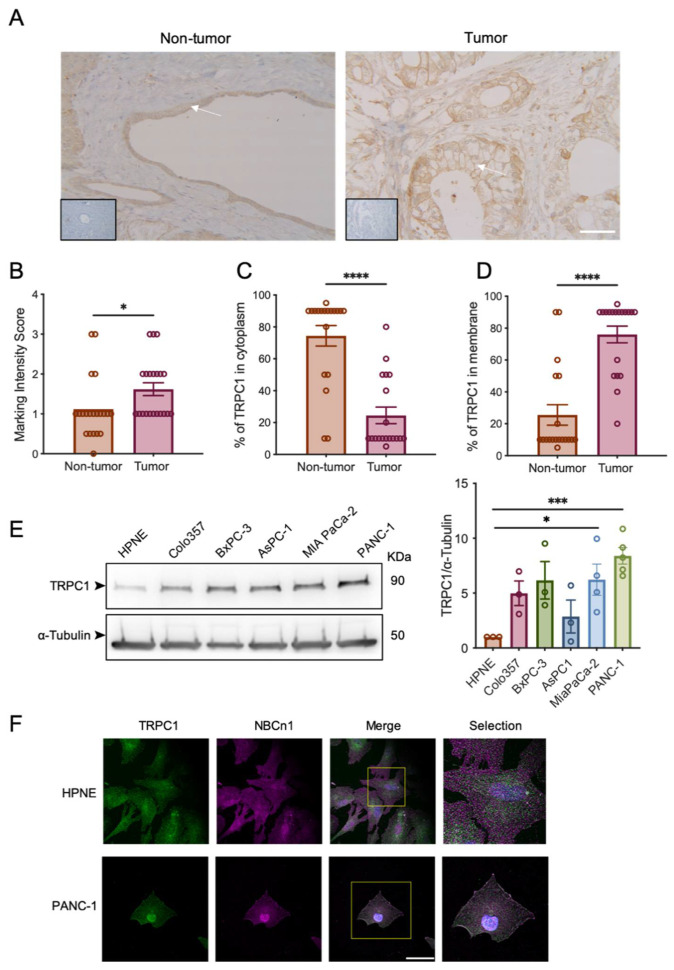
TRPC1 is overexpressed in PDAC tissue and cell lines, where it localizes to the plasma membrane. (**A**) Representative images of TRPC1 expression in human PDAC tissue, performed by immunohistochemistry (IHC). White arrows indicate expression in the cytoplasm (left panel) and the plasma membrane (right panel). Inserts represent negative controls, scale bar = 40 µm. (**B**) Quantification of TRPC1 marking intensity score between non-tumor and tumor tissue (N = 21). (**C**) Quantification of TRPC1 expressed in the cytoplasm and (**D**) membrane (N = 21). (**E**) Western blot analysis (left panel) and quantification (right panel) of TRPC1 expression in commercial-available PDAC cell lines (*n* = 3–5). (**F**) Representative immunofluorescent analysis of TRPC1 in HPNE and PANC-1 cells (*n* = 3), scale bar = 20 µm. Welch’s correction t-test was used to determine the significant difference between control and tumor conditions. *, *** and **** indicate *p* < 0.05, 0.01, 0.001, and 0.0001, respectively.

TRPC1 is located both in the plasma membrane and intracellular sites [39]. However, its localization in pancreatic ductal cells and PDAC cells is still unknown, although TRPC1 has been found in the apical and lateral regions of the basolateral membrane of pancreatic acinar cells [40]. Thus, we compared the TRPC1 expression and localization in human PDAC and adjacent non-tumor tissue samples from our local cohort (N = 21). Our immunohistochemistry (IHC) results confirmed the GEPIA2 analysis, showing a significant upregulation of TRPC1 by 33 ± 12.5% (*p* < 0.05, Figure 2A,B). Moreover, TRPC1 was considerably more localized to the plasma membrane in tumor cells when compared to non-tumor cells, where TRPC1 was found mainly expressed in the cytoplasm (*p* < 0.0001, Figure 2A,C,D). Transcriptional expression of TRPC1 has been found in different types of commercially available PDAC cell lines (BxPC-3, CAPAN-1, CFPAC, and PANC-1) [41,42]. However, to our knowledge, its expression has not yet been compared to a normal pancreatic cell line. We investigated the protein expression of TRPC1 in different pancreatic cell lines. TRPC1 was overexpressed in five different PDAC cell lines, significantly in the MIA PaCa-2 cell line by 83 ± 22.6% and in the PANC-1 cell line by 88 ± 8.9% when compared to the normal-like pancreatic ductal cell line HPNE (*n* = 4–5, *p* < 0.05, Figure 2E). Since the expression of TRPC1 was significantly upregulated in the particularly aggressive PDAC cell line PANC-1, this cell line was the focus of the subsequent study. Hence, we investigated the localization of TRPC1 in PANC-1 and HPNE cells. We found a robust membrane localization of TRPC1 in PANC-1 cells, which we did not find in HPNE cells, where it seems that TRPC1 tended to be localized in the cytoplasm (Figure 2F). Collectively, these findings suggest that TRPC1 is upregulated in human PDAC tissue and cell lines when compared to adjacent non-tumor tissue and a non-cancerous cell line. TRPC1 also localizes to the plasma membrane of tumor cells when compared to non-tumor cells.

### 2.3. The Knockdown of TPRC1 Inhibits PANC-1 Cell and Spheroid Growth

**Figure 3:**
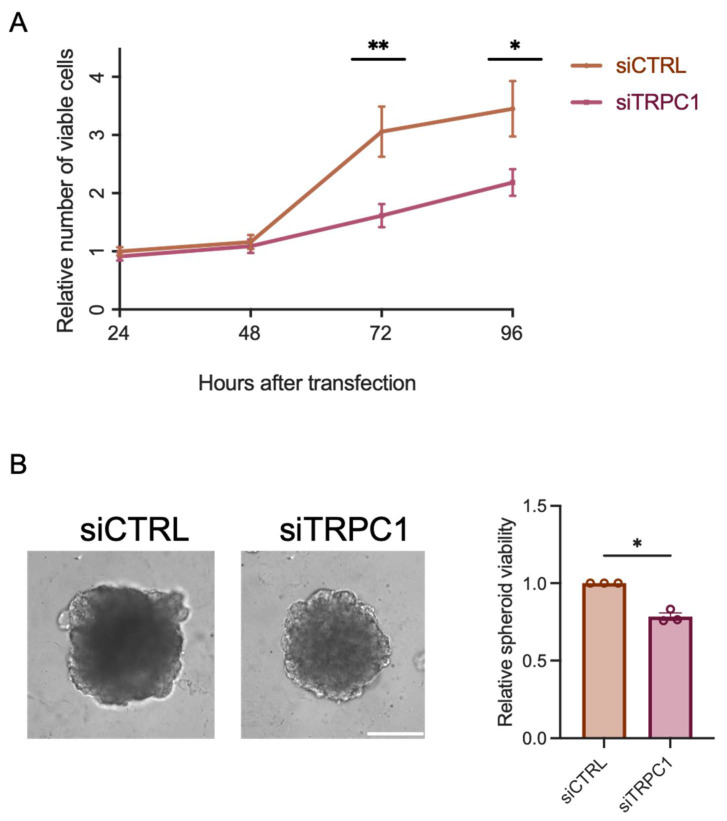
The knockdown of TRPC1 inhibits PANC-1 cell proliferation and spheroid growth. (**A**) trypan blue analysis of transfected PANC-1 cells shows the relative number of viable cells compared to siCTRL 24, 48, 72, and 96 h post-transfection (*n* = 4). (**B**) Representative images of PANC-1 spheroids grown for nine days (left panel) and quantification of CellTiter-Glo^®^ assay (right panel) (*n* = 3), scale bar = 400 µm. Welch’s correction t-test was used to determine the significant difference between siCTRL and siTRPC1; * and ** indicate *p* < 0.05 and 0.01, respectively.

To study the role of TRPC1, we validated our knockdown (KD) model (Supplementary Figure S1). TRPC1 protein expression decreased by 44 ± 10% after 72 h of siRNA transfection against TRPC1. Similar results were obtained by assessing the TRPC1 transcriptional levels 48, 72, and 96 h post-transfection (Supplementary Figure S1A). We investigated the role of TRPC1 in PANC-1 cell proliferation by the trypan blue assay. The KD of TRPC1 resulted in a significant decrease of PANC-1 cell viability by 48 ± 17.4% and 38 ± 17.6% after 72 and 96 h post-transfection, respectively (*n* = 4, *p* < 0.05, Figure 3A). Even though our trypan blue assay indicated increased mortality 48 h post-transfection, our annexin-5 analysis did not show any significant difference in cell mortality between conditions (Supplementary Figure S2A–C). As 3D models imitate in vivo cell–cell and cell–extracellular matrix interactions better than traditional two-dimensional (2D) cell cultures [43], we established a 3D model of PANC-1 cell spheroids grown for nine days upon a KD of TRPC1. First, we confirmed the KD of TRPC1 after nine days, where TRPC1 protein levels decreased by 22 ± 1.0% (Supplementary Figure S1C). This KD of TRPC1 significantly decreased the viability of spheroids after nine days by 22 ± 2.3% (*n* = 3, *p* < 0.05, Figure 3B). Collectively, these results suggest that silencing of TRPC1 inhibits PANC-1 cell and spheroid proliferation.

### 2.4. The Knockdown of TRPC1 Regulates Cell Cycle Progression by Reducing the Expression of CDK6, 2, and Cyclin A and Increasing the Expression of p21^CIP1^

**Figure 4:**
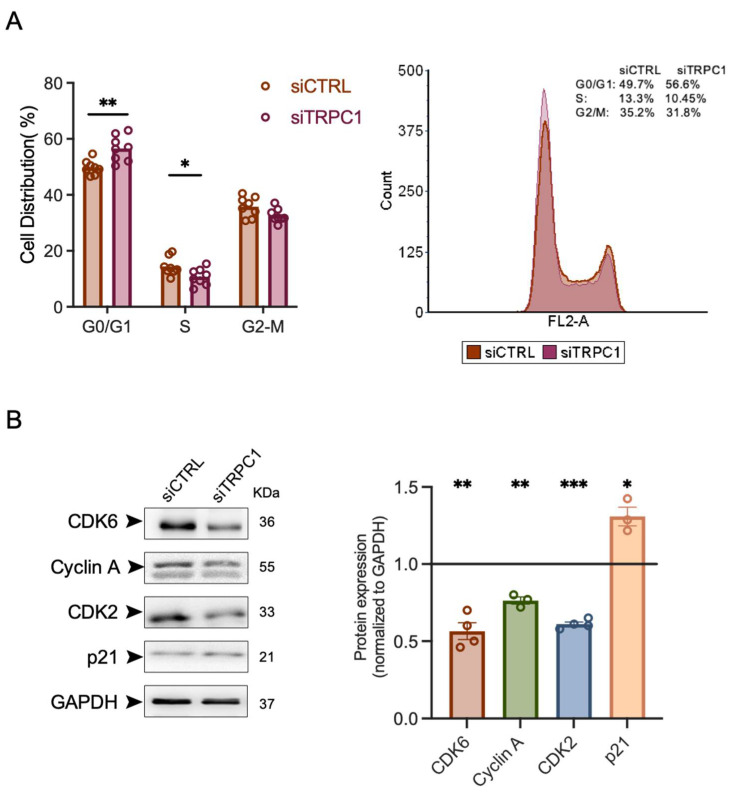
The knockdown of TRPC1 arrests PANC-1 cells in G1/S phases, decreases the expression of CDK6, 2, Cyclin A, and increases the expression of p21^CIP1^. (**A**) Quantification of cell cycle analysis representing the percentage of cells in each cell cycle phase of both transfected PANC-1 cells with either siCTRL or siTRPC1 (left panel) and representative data from FACS acquisition (generated with FCS Express 7) (right panel) (*n* = 4). (**B**) Western blot analysis of relevant cyclin-dependent kinase complexes and cyclins and their inhibitor p21^CIP1^ (left panel). Quantification of western blot analysis representing the expression of proteins in siTRPC1 lysates compared to siCTRL lysates (right panel) (*n* = 3–4). Welch’s correction t-test was used to determine the significant difference between siCTRL and siTRPC1. *, ** and *** indicate *p* < 0.05, 0.01 and 0.001, respectively.

To investigate the possible mechanism by which the KD of TRPC1 affects PANC-1 cell proliferation, the cell cycle distribution was examined by flow cytometry. We found that the KD of TRPC1 increased the number of PANC-1 cells in G0/G1 phase by 13.8 ± 3.7% and decreased the number of cells by 25.7% ±10.3% in S-phase (*n* = 4, *p* < 0.05, Figure 4A). To further confirm the TRPC1-dependent regulation of cell cycle progression, we analyzed the expressions of several essential cell cycle-driven proteins involved in the G1- and S-phases. The immunoblot analysis showed that the expressions of CDK6 and CDK2 were significantly decreased by 44 ± 5.3%, 39 ± 1.4%, and Cyclin A by 24 ± 2.3%, respectively, upon KD of TRPC1 (*n* = 3–4, *p* < 0.05, Figure 4B). Moreover, the expression of p21^CIP1,^ known to inhibit the CDK2 and CDK6 complexes, was significantly upregulated by 23 ± 4.6% (*n* = 3, *p* < 0.05, Figure 4B). However, TRPC1 KD failed to affect the expression of cyclin B1, D1, D3, and E, along with CDK1 and CDK4 (Supplementary Figure S3). Together, this suggests that TRPC1 depletion arrests cells in the G1 phase and decreases the cell fraction in the S-phase.

### 2.5. The Knockdown of TRPC1 Does Not Affect Ca^2+^ Entry nor Store-Operated Ca^2+^ Entry and Decreases Cell Proliferation Independently of Extracellular Ca^2+^

**Figure 5:**
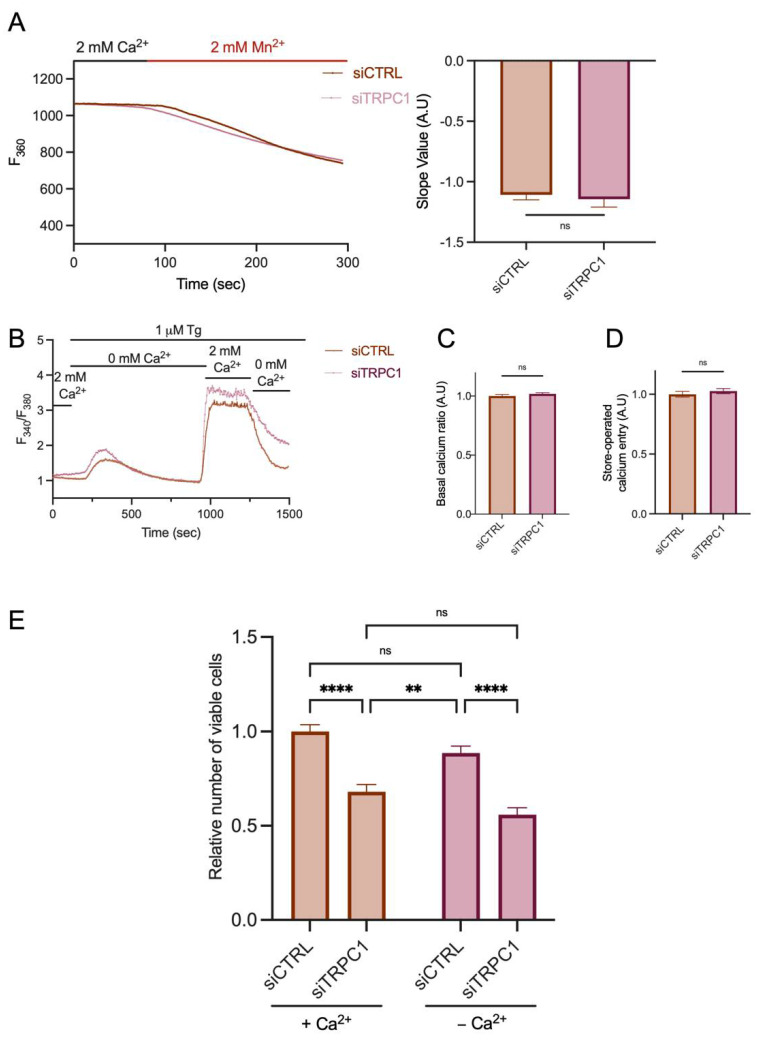
The knockdown of TRPC1 does not affect Ca^2+^ entry and the proliferative role of PANC-1 cells does not depend on extracellular Ca^2+^ concentration. (**A**) Representative traces of Mn^2+^ quenching in PANC-1 cells (left panel) and the quantification of siCTRL and siTRPC1 transfected PANC-1 cells (right panel) (number of analyzed cells siCTRL = 342 and siTRPC *n* = 382). ns indicates non-significant. (**B**) Representative traces of the classical store-operated Ca^2+^ entry protocol. Cells were perfused with 2 mM Ca^2+^ for 1 min, then with 0 mM Ca^2+^ and 1 µM thapsigargin (Tg) for 12 min, followed by 2 mM Ca^2+^ for 5 min, and finally perfused with 0 mM Ca^2+^. ns indicates non-significant. (**C**) Quantification of basal Ca^2+^ ratio (0 mM Ca^2+^) in siCTRL and siTRPC1 transfected PANC-1 cells (number of analyzed cells siCTRL = 303 and siTRPC1 = 280). ns indicates non-significant. (**D**) Quantification of SOCE (2 mM Ca^2^ after internal Ca^2+^-store depletion) in siCTRL and siTRPC1 transfected PANC-1 cells (number of analyzed cells siCTRL = 303 and siTRPC *n* = 280). (**E**) Trypan blue assay analysis of siCTRL and siTRPC1 transfected PANC-1 cells (for 72 h) either treated with medium containing extracellular Ca^2+^ concentrations (+Ca^2+^), or with medium depleted for extracellular Ca^2+^ (−Ca^2+^), for 48 h (*n* = 3). Welch’s correction t-test and Tukey’s multiple comparison test (**E**) were used to determine significant difference between siCTRL and siTRPC1 conditions. **, and **** indicate *p* < 0.01, and 0.0001, respectively.

Although it has been shown that TRPC1 functions as a SOC channel in the PDAC cell line BxPC-3 [42], it is very controversial whether TRPC1 is a modulator of Ca^2+^ entry [44]. In different colorectal, thyroid, and hepatocellular cancer cells, TRPC1 did not contribute to cancer progression through SOCE [13,24,37,45]. Therefore, we investigated the role of TRPC1 in Ca^2+^ entry of PANC-1 cells using the manganese-quenching technique. TRPC1 silencing did not affect the Mn^2+^ quench (6 ± 9.2% increase, *n* = 342 and 382, for siCTRL and siTRPC1, respectively, Figure 5A). Furthermore, using the classical SOCE protocol, we showed that TRPC1 silencing did not participate in SOCE (2.6 ± 3.2% increase, *n* = 303 and 280, for siCTRL and siTRPC1, respectively, Figure 5B,C) or affected the intracellular Ca^2+^ concentration estimated by the basal ratio (1.8 ± 1.7% increase, *n* = 303 and 280, for siCTRL and siTRPC1, respectively, Figure 5B,D). As TRPC1 does not appear to be involved in Ca^2+^ entry in PANC-1 cells, we investigated whether PANC-1 cell proliferation was Ca^2+^-dependent or independent. We evaluated the proliferation of PANC-1 cells cultured for 48 h in a medium depleted of Ca^2+^. This extracellular Ca^2+^ chelation by EGTA did not decrease cell viability (Figure 5E). Moreover, TRPC1 silencing further decreased the cell proliferation regardless of the extracellular Ca^2+^ concentration by 32 ± 5.3% and 37.0 ± 5.2%, with or without Ca^2+^, respectively (*n* = 3, *p* < 0.01). These results suggest that TRPC1 is not involved in the Ca^2+^ entry of PANC-1 cells and regulates cell proliferation by a Ca^2+^-independent mechanism.

### 2.6. TRPC1 Strongly Regulates AKT Phosphorylation in PANC-1 Cells

**Figure 6:**
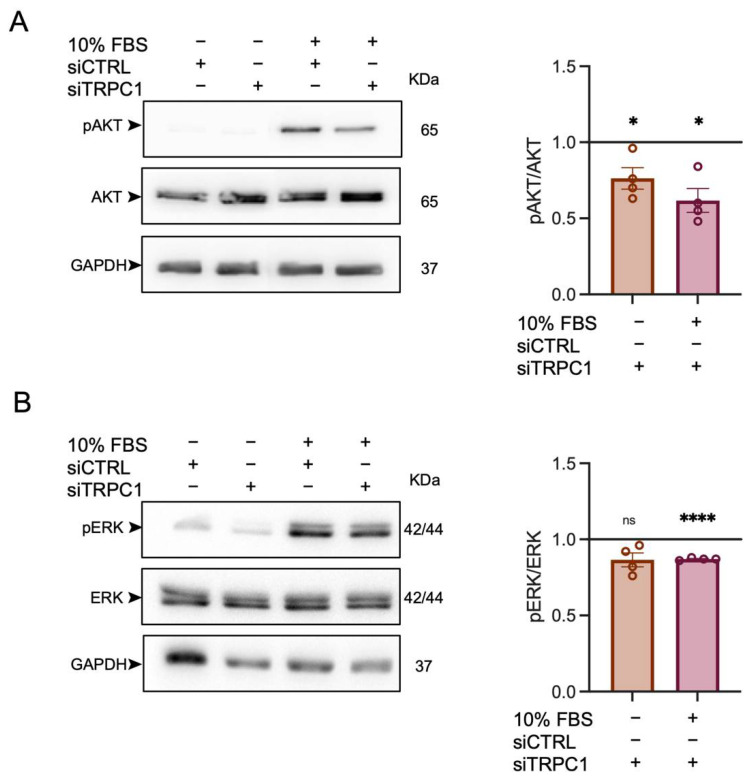
The knockdown of TRPC1 decreases PANC-1 cell proliferation through the phosphorylation of AKT pathways. (**A**) Western blot analysis of phosphorylated AKT (pAKT) and total AKT in transfected PANC-1 cells after mitogen-activation with FBS for either 0 or 30 min (left panel). Quantification of the western blot analysis compared to siCTRL either after 0 min or 30 min of mitogen activation (right panel). * indicates *p* < 0.05. (**B**) Western blot analysis of phosphorylated ERK1/2 (pERK1/2) and total ERK in transfected PANC-1 cells after mitogen-action for either 0 or 30 min (left panel). Quantification of the western blot analysis compared to siCTRL either after 0 min or 30 min of mitogen activation (right panel). Welch’s correction *t*-test was used to determine the significant difference between siCTRL and siTRPC1 conditions. ns indicate non-significant and **** *p* < 0.0001, respectively.

Two key downstream proteins responsible for cell proliferation are the serine/threonine kinases AKT and ERK1/2 [46]. The phosphorylation of these kinases has been shown to be dependent on TRPC1 expression [28,47]. We investigated the role of TRPC1 in the phosphorylation of AKT and ERK1/2 in transfected PANC-1 cells starved for 24 h, which were then mitogen-activated with FBS for 30 min. The immunoblot analysis revealed that the KD of TRPC1 significantly reduced AKT activation by 39 ± 7.8%, while total AKT expression remained unchanged (*n* = 4, *p* < 0.05, Figure 6A). A slight reduction of ERK1/2 phosphorylation (13 ± 0.4%, (*n* = 4, *p* < 0.0001, Figure 6B) was observed in cells transfected with siTRPC1. These results indicate that TRPC1 regulates PANC-1 cell proliferation through activation of AKT.

### 2.7. TRPC1 Interacts with PI3K, Which Is Attenuated upon the Knockdown of TRPC1

**Figure 7:**
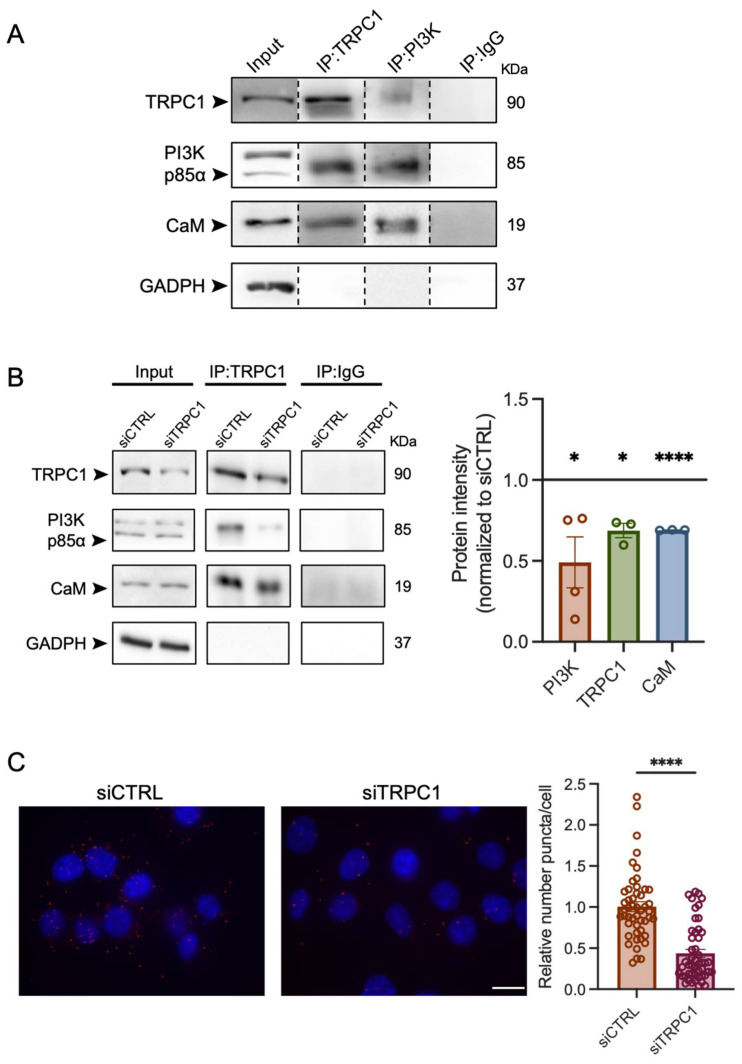
TRPC1 forms a complex with the PI3K p85α subunit and CaM, which is abolished upon TRPC1 knockdown. (**A**) Representative western blot analysis of co-immunoprecipitation of TRPC1 and PI3K p85α subunit with CaM in non-transfected PANC-1 cells. (**B**) Representative western blot analysis of co-immunoprecipitation of TRPC1 with PI3K p85α and CaM in transfected PANC-1 cells (left panel). Quantification of western blot analysis compared to siCTRL represents the effect of TRPC1 KD on the protein interaction (right panel) (*n* = 3–4). * and **** indicate *p* < 0.05 and 0.0001, respectively. (**C**) Representative images of proximity ligation assay (PLA) in transfected PANC-1 cells (left panel). Quantification of PLA where siTRPC1 is compared to the relative number of siCTRL (*n* = 3. At least 20 images were analyzed for each experiment, see material and methods for details) (right panel). **** indicates *p* < 0.0001, respectively.

TRPC1 has been shown to be involved in activating the PI3K signaling pathway [13,38,47]. In addition, it has been demonstrated that CaM is a connexin between TRPC1 and the p85α subunit of PI3K [13]. Thus, we investigated whether TRPC1 interacts with the PI3K-p85α subunit in PANC-1 cells using Co-IP and PLA techniques. Our results by PLA analysis showed that TRPC1 and PI3K are located particularly close to each other (<40 nm) and can possibly interact. There was a relative decrease of 57 ± 7.8% puncta per cell in siTRPC1 cells compared to siCTRL cells (*n* = 3, *p* < 0.0001, Figure 7C). By co-IP, we found that TRPC1 interacts with PI3K and CaM in PANC-1 cells (Figure 7A). As an outcome of TRPC1 silencing, the interaction between TRPC1, PI3K, and CaM was reduced (Figure 7B) by 32 ± 4.5%, 51 ± 15.8%, and 31 ± 0.1%, respectively. These results suggest that the loss of TRPC1 can attenuate PI3K signaling.

## 3. Discussion

A wide range of physiological functions has been attributed to the expression and regulation of TRPC1, including cell proliferation, migration, invasion, and apoptosis [9,10,48,49]. In recent years, there has been increased interest in TRPC1 and its connection with tumor-related functions, and several studies have reported different downstream mechanisms of TRPC1 in several types of cancers [11]. However, there is sparse evidence of its involvement in PDAC progression. In this study, we revealed the role of TRPC1 and its downstream mechanisms that contribute to PDAC cell proliferation. TRPC1 is upregulated in various types of cancer, such as tongue, breast, gastric, ovarian, colorectal, and renal cell carcinoma, where its expression correlates with different clinical factors [12,13,14,15,16,17,18,19,20,21]. For instance, the high expression of TRPC1 correlated with low overall and event-free survival in colorectal cancer patients [13,17] and disease-free survival in lung cancer patients [21]. In endometrial cancer patients, the high expression of TRPC1 was correlated with low overall survival, even though the expression of TRPC1 was downregulated when comparing endometrial cancer with adjacent tissue samples [15]. In congruence with this, we show that TRPC1 is upregulated in PDAC tissue compared to non-tumor tissue samples. Interestingly, the expression of TRPC1 is higher in the basal-like subtype of PDAC, which is associated with a poorer clinical outcome and loss of differentiation [50]. In addition, our findings show that the high expression of TRPC1 correlated with low overall survival in PDAC patients. However, the dysregulated expression of TRPC1 will remain controversial and seems to be cell type-specific, as the upregulation of TRPC1 appears to have a protective role in prostate [51,52] and some breast cancer patients [33]. Consistent with the increased TRPC1 mRNA expression, TRPC1 protein expression also significantly increased in human PDAC samples, where TRPC1 localizes to the plasma membrane, compared to adjacent non-tumor tissue, where it localizes in the cytoplasmic compartments.

In addition to expression in human tissue, TRPC1 has been shown to be upregulated in cancer cell lines compared to a corresponding non-cancerous cell line. For instance, TRPC1 is upregulated in breast cancer cell lines [33] and colorectal cancer cell lines [13]. To our knowledge, the expression of TRPC1 has not yet been evaluated in commercially-available PDAC cell lines compared to a duct-like control cell line. Interestingly, TRPC1 expression markedly increased in the more aggressive PDAC cell line PANC-1, and the cell line was the focus of the subsequent study. Remarkably, in PANC-1 cells, the localization of TRPC1 is in the cell membrane when compared to the HPNE cells, where TRPC1 seems to be located in the cytoplasm.

To investigate the role of TRPC1 in PDAC proliferation and growth, we performed loss of function experiments in 2D and 3D cell models. We found that TRPC1 silencing inhibited 2D cell proliferation and spheroid growth. The same effect has been found in neuroblastoma [22], glioblastoma [23], thyroid [24], breast [12,25,26,27], lung, [28], liver [29,30], ovarian [31], and colon cancer [13]. This indicates that the upregulation of TRPC1 stimulates proliferation in several cancer types. Repressed cell proliferation is often associated with disruption of cell cycle progression by the dysregulation of cyclins and cyclin-dependent kinase (CDK) complexes [53]. In this study, the KD of TRPC1 accumulated cells in the G0/G1 phase reduced the number of cells in the S-phase and the expressions of CDK6, CDK2, Cyclin A, and increased p21^CIP1^ expression. These proteins are essential in regulating progress through the G1/S phases of the cell cycle. Such a scenario has also been reported for other cancer cell models, for instance, in A549 lung cancer cells, where the KD of TRPC1 decreased the protein expression of Cyclin D1 and D3, leading to an increased number of cells in the G1 phase [28]. In addition, MCF-7 breast cancer cells accumulated in the G1 phase upon the KD of TRPC1 [12], which was also found in the thyroid cancer cell line ML-1, where the expression of the CDK6, Cyclin D2, and D3 decreased, and the expression of their regulating inhibitor p21 increased [24]. This indicates that TRPC1 is important for different cancer cell types to progress through the G1/S phases and that it is cell type-specific how CDK/Cyclin complexes regulate this progression. 

The pathophysiological importance of TRPC1 in the plasma membrane is often associated with its role in Ca^2+^ entry, as the Ca^2+^ flux has been shown to be essential for tumor progression [11]. However, it is controversial whether TRPC1 regulates relevant cellular processes of tumorigenesis through Ca^2+^ as several studies have shown that these events can be regulated by TRPC1 independently of Ca^2+^ entry [13,24,30,34,35,36]. Even though SOCE and constitutive Ca^2+^-entry have been shown to regulate cell proliferation through cell cycle progression, it is well accepted that transformed cells can proliferate independently from Ca^2+^-entry [5,7,13,24]. This is consistent with our findings, where the KD of TRPC1 did not affect Ca^2+^-entry, SOCE, which was slightly but not significantly increased, and the chelation of Ca^2+^ had no effect on PANC-1 cell proliferation, suggesting that the PANC-1 cell and spheroid proliferation is Ca^2+^ independent.

It is well known that the PI3K and MAPK signaling pathways play essential roles in cell proliferation and growth [54]. Inhibition of these proteins can lead to cell cycle arrest in PDAC cell lines [55]. Our findings show that KD of TRPC1 decreases the phosphorylation of AKT and, to a lesser extent, ERK1/2, which could explain the decrease of cell cycle regulating proteins and cell cycle arrest in the G1/S phases. This indicates that the PI3K signaling axis profoundly regulates PANC-1 cell proliferation through TRPC1. Hence, we investigated the physical interaction of TRPC1 and PI3K and its associated connecting protein CaM. It is known that TRPC channels can bind to CaM and thereby regulate physiological and pathophysiological processes [9]. In addition, CaM can bind to the PI3K p85α subunit, which removes its inhibitory effect on the catalytic subunit p110, and sequentially activates PI3K signaling [56]. The physical interaction between TRPC1 and PI3K has been shown to regulate myoblast differentiation [57], and only one other study besides ours shows the interaction between TRPC1, PI3K, and CaM [13]. However, a similar interaction of CaM and PI3K with TRPC6 has been found to regulate bovine aortic endothelial cell migration [58]. Here, we found that abolishing TRPC1 decreased the interaction between these proteins. As the phosphorylation of AKT was significantly reduced upon KD of TRPC1, our findings suggest that TRPC1 regulates PANC-1 cell proliferation by forming a complex with PI3K and CaM, thereby activating downstream signaling. However, some studies have shown that KD of TRPC1 decreased SOCE and thereby AKT phosphorylation in different cancer cell models [28,29]. Our results show that the phosphorylation of AKT seems to be Ca^2+^-independent. This finding is in congruence with other reports, where the KD of TRPC1 decreased the phosphorylation of AKT in a Ca^2+^-independent manner. For instance, in the PTEN deficient breast cancer cell line MDA-MB-468, TRPC1 silencing reduced the phosphorylation of AKT even though it slightly increased SOCE levels [36,47]. The same was found in some colorectal cancer cell lines, where it was proposed that TRPC1 regulated the PI3K/CaM/AKT axis independently of Ca^2+^ [13].

In conclusion, we show for the first time that TRPC1 is upregulated in aggressive subtypes of PDAC patient tumor tissue and cell lines. TRPC1 regulates PDAC cell and spheroid proliferation through the direct interaction with the PI3K and CaM, thereby activating downstream signaling events implicated in G1 phase progression and G1/S transition, likely through a Ca^2+^-independent mechanism. 

## 4. Materials and Methods

### 4.1. Public Database Analysis

TRPC1 mRNA expression was investigated by using online available datasets. Whisker boxplots of TRPC1 mRNA expression were generated using GEPIA2 ttp://gepia2.cancer-pku.cn/, accessed on 1 June 2021) that compiles Genotype-Tissue Expression (GTEx) (http://www.GTEXportal.org/, accessed on 1 June 2021), and The Cancer Genome Atlas (TCGA) (http://www.cancergenome.nih.gov/, accessed on 1 June 2021) datasets of normal and tumoral samples from different organs of interest, in our case pancreas and PDAC (PAAD). Survival analysis was performed using the SurvExpress online tool (available at http://bioinformatica.mty.itesm.mx/SurvExpress, accessed on 2 March 2022), as previously described [59]. 

### 4.2. Immunohistochemistry in Human PDAC Samples

All experimental protocols were approved by the ethical committee of the University Hospital Center of Amiens (reference: PI2021_843_0027). Human tissue samples from PDAC patients were obtained and processed at the University Hospital Center of Amiens. Classical immunohistochemistry was performed from routinely processed formalin-fixed paraffin-embedded PDAC tumor material. First, 4 µM thick sections were deparaffinized in xylene and then rehydrated in ethanol. Then, the endogenous peroxidase activity was blocked before the antigen retrieval. The cell conditioning solution CC1 (BenchMark XT, Ventana, Rotkreuz, Switzerland) was used for antigen retrieval. Automatic immunohistochemical staining was carried out on a BenchMark ULTRA system (Ventana, Rotkreuz, Switzerland) using antibodies against TRPC1 (1/50, Abcam, Waltham, MA, USA). Next, the biotin-labeled secondary antibody was applied, followed by the addition of avidin–biotin–peroxidase complex treatment. Reactions were developed using a chromogenic reaction with 3,3′-diaminobenzidine tetrahydrochloride (iVIEW DAB Detection Kit, Ventana). The tissue sections were counterstained with hematoxylin. The TRPC1 antibody was certified for immunohistochemical use by the manufacturer. Negative controls were performed with the same technique and conditions, without the addition of the primary antibody. Histological examination and quantitative evaluation were performed in cooperation with a pathologist (HS, PR), using a Leica inverted microscope. The marking intensity score ranged from 0 to 3 (0 = no immunostaining, 1 = weak immunostaining, 2 = moderate immunostaining, 3 = strong immunostaining). The TRPC1 localization was evaluated as a percentage in the cytoplasm or membrane.

### 4.3. Cell Culture

The hTERT-immortalized Human Pancreatic Nestin-Expressing cells (HPNE) were purchased from the American Type Culture Collection (ATCC, Molsheim, France). HPNE were grown in 75% DMEM without glucose (Sigma, Cat no. D-503, St. Louis, MO, USA), 25% Medium M3 Base (Incell Corp., San Antonio, TX, USA, Cat no. M300F-500), which were completed with 10% fetal bovine serum (Cat no. P30-3031, PAN Biotech, Aidenbach, Germany), 5.5 mM glucose and 750 ng/mL puromycin. Colo357, BxPC-3, AsPC-1, MIA PaCa-2, and PANC-1 cells were kindly provided by Prof. Anna Trauzold (Institute of Experimental Cancer Research, Kiel University, Kiel, Germany). These cell lines were grown in RPMI-1640 (Sigma-Aldrich, Cat no. R8758), supplemented with 10% fetal bovine serum (Cat no. P30-3031, PAN Biotech), 1 mM sodium pyruvate (Gibco, Waltham, MA, USA), and 1% Glutamax (Gibco). Cells were grown at 37 °C, 95% humidity, 5% CO2, and passaged with trypsin-EDTA 0.25% (Sigma, Saint-Quentin-Fallavier, France) when cells reached a confluency of 70–80%. Cell cultures were not used for more than 20 passages.

### 4.4. Transient Transfections

Cells were transfected with small interfering RNA (siRNA) by electroporation using nucleofection (Amaxa Biosystems, Lonza, Aubergenville, France). PANC-1 cells (1× 10^6^) were transiently nucleofected according to the manufacturer’s protocol with 4 μg of scrambled siRNA (siCTRL, duplex negative control, Eurogentec) or with siRNA directed against TRPC1, (siTRPC1, ON-TARGET plus SMART pool siRNA, Dharmacon Research, Chicago, IL, USA). All the experiments were performed 72 h after the siRNA transfection.

### 4.5. 3D-Spheroid Growth and CellTiter Glo Assay

A total of 2000 transfected PANC-1 cells were seeded per well in round-bottomed, ultra-low attachment 96-well plates (Corning, NY, USA) in 200 μL growth medium, supplemented with 2% GelTrex LDEV-Free reduced growth factor basement membrane matrix (ThermoFisher Scientific, Waltham, MA, USA). Cells were subsequently spun down at 750 RCF for 20 min and were grown for 9 days at 37 °C with 95% humidity and 5% CO_2_. 100 μL medium was exchanged every second day. Light microscopic images of the spheroids at 10× magnification were acquired on days 2, 4, 7, and 9. On day 9, spheroids (in a replicate of 3) in 100 µL medium were transferred to a black 96-well plate where 100 µL CellTiter-Glo^®^ 3D Reagent (Promega, Madison, WI, USA) was added. The black plate was wrapped in aluminum foil, shaken for 5 min, and then incubated for 25 min at room temperature. The luminescence signal was recorded using a FLUOStar Optima microplate reader (BMG Labtech, Ortenberg, Germany).

### 4.6. Western Blot Analysis

Proteins were extracted and separated as previously described [60]. The primary antibodies used were: anti-TRPC1 (1:1000, Abcam, Waltham, MA, USA), anti-αTubulin (Sigma-Aldrich, France), anti-CDK6 (1:500, Cell Signaling Tech., Danvers, MA, USA), anti-Cyclin A (1:500, Santacruz Biotechnology, Dallas, TX, USA), anti-CDK2 (1:500, Cell Signaling Tech., Danvers, MA, USA), anti-p21 (1:500, Cell Signaling Tech., Danvers, MA, USA), anti- GAPDH (1:2000, Cell Signaling Tech., Danvers, MA, USA), anti-PI3K p85α (1:500, Bioworld Technology), and anti-Calmodulin (1:500, Santacruz Biotechnology, Dallas, TX, USA). Secondary antibodies were coupled to horseradish peroxidase, permitting protein detection with an enhanced chemiluminescence kit (Ozyme, Saint-Cyr-l’Ecole, France). Quantification was performed with the ImageJ analysis tool. All experiments were normalized to α-Tubulin or GAPDH, which were used as reference proteins.

### 4.7. Co-Immunoprecipitation

A total of 500 µg of proteins from non-transfected or transfected PANC-1 cells were used for co-immunoprecipitation with SureBeads™ Protein A Magnetic Beads (Biorad, France). First, the beads were washed thoroughly, as described in the manufacturer’s protocol. Then 1 µg of either TRPC1 antibody (Abcam, Waltham, MA, USA) or PI3K p85α antibody was resuspended with the beads for 30 min. After another sequential washing step, protein lysates were added to the beads and slowly rotated for 2 h at room temperature. The beads were washed and eluted according to the manufacturer’s protocol. After denaturation, proteins were used for a standard Western blot, as described above. For input, 50 µg of proteins from the corresponding co-immunoprecipitation samples were used. 

### 4.8. Trypan Blue Assay

The 8 × 10^4^ PANC-1 cells were seeded in 35 mm Petri-dishes for 72 h after siRNA transfection. Cells were washed in PBS, trypsinized, and diluted (1:5) in trypan blue solution (Sigma-Aldrich, France), then counted six times using the standard Malassez cell method. Proliferation was calculated as the total number of viable cells (cells alive/white cells), normalized to the control, and the mortality was calculated as the percentage of dead cells (blue cells), compared to the control. To test the effect of Ca^2+^ on proliferation, the same procedure as described above was carried out, but after 24 h of seeding, the medium was changed to medium with standard conditions containing 1 mM Ca^2+^ (referred to as conditions with Ca^2+^) or containing Ethylene Glycol Tetraacetic Acid (EGTA), to chelate Ca^2+^ and to end with a final concentration of 30 µM (referred to conditions without Ca^2+^). Therefore, cells were transfected for 72 h, and Ca^2+^ where chelated for 48 h.

### 4.9. Flow Cytometry

Flow cytometry was carried out on ethanol-fixed cells by a propidium iodide assay. Duplicates of 2 × 10^5^ transfected PANC-1 cells were seeded in 60 mm Petri dishes and collected after 72 h. Cells were washed in PBS, trypsinized, collected in PBS + EDTA (5 mM), and fixed with cold absolute ethanol (≥99.8%, Sigma-Aldrich) for at least 6 h, at 4 °C. Then, cells were pelleted, resuspended in PBS + EDTA (5 mM), treated with 20 µg/mL RNase A (Sigma-Aldrich) for 30 min at room temperature, and stained with 50 µg/mL of propidium iodide (Sigma-Aldrich, St. Quentin Fallavier, France). Samples were then analyzed by a flow cytometer (Accuri^®^, Dominique Deutscher, Brumath, France), and the cell percentage in different phases was calculated using Cyflogic software and illustrated with FCS Express 7.

### 4.10. Calcium Imaging

Ca^2+^ imaging acquisition was performed as previously described [61]. Briefly, 25 × 10^3^ transfected PANC-1 cells were seeded on glass coverslips 72 h before each experiment. Then, cells were incubated with 3.33 μM Fura-2/AM (Sigma, Saint-Quentin-Fallavier, France) at 37 °C in the dark for 45 min. Cells on the coverslip were washed twice with extracellular saline solution (145 mM NaCl, 5 mM KCl, 2 mM CaCl_2_, 1 mM MgCl_2_, 5 mM glucose, 10 mM HEPES, pH 7.4), and placed on the stage of a fluorescence microscope (Axiovert 200; Carl Zeiss, Oberkochen, Germany). Here, cells were excited at 340 and 380 nm using a monochromator (polychrome IV, TILL Photonics, Gräfelfing, Germany), and fluorescent emission was captured with a Cool SNAPHQ camera (Princeton Instruments, Lisses, France) after filtration through a long-pass filter (510 nm). Metafluor software (version 7.1.7.0, Molecular Devices, San Jose, CA, USA) was used for signal acquisition and data analysis. During acquisition, cells were continuously perfused with the saline solution. Store-operated Ca^2+^ entry (SOCE) was triggered by applying the classical protocol, first by perfusing 2 mM Ca^2+^ (for 1 min), then 0 mM Ca^2+^ during 1 min followed by 1 μM thapsigargin in 0 mM Ca^2+^ for Ca^2+^ store depletion for 12 min, followed by perfusion of 2 mM Ca^2+^ (for 5 min). The intracellular Ca^2+^ concentration is derived from the ratio of emitted fluorescence intensities for each of the excitation wavelengths (F340/F380). To estimate divalent cation influx under basal conditions, we used the manganese (Mn^2+^) quenching technique. After Fura2/AM loading, cells were washed and excited at 360 nm, where fluorescence was recorded at 510 nm. After 1.5 min, the Ca^2+^ (2 mM) present in the saline solution was replaced by 2 mM Mn^2+^ solution. The Mn^2+^ quenching extracellular solution was composed of 145 mM NaCl, 5 mM KCl, 2 mM MnCl_2_, 1 mM MgCl_2_, 5 mM glucose, and 10 mM HEPES (pH 7.4). The Mn^2+^ influx, a corroborate of the Ca^2+^ influx, was estimated from the quenching rate of fluorescence at 360 nm by calculating the slope.

### 4.11. Immunofluorescence

The 8 × 10^4^ non-transfected HPNE or PANC-1 cells were seeded on coverslips. After 48 h, cells were washed in cold PBS fixed for 20 min at room temperature in 4% paraformaldehyde (PFA, Sigma-Aldrich) and washed twice in PBS. Cells were permeabilized in 0.1% Triton^TM^X-100 (Sigma, Saint-Quentin-Fallavier, France) for 10 min and blocked in 5% Bovine Serum Albumin (BSA, Sigma Aldrich no. A7906) for 30 min. Primary antibodies (anti-TRPC1, 1:100, Santacruz Biotechnology, Dallas, TX, USA, NBCn1 was used as a membrane marker, 1:400, Abcam, Waltham, MA, USA) were added overnight at 4 °C and secondary antibodies for 1 h at room temperature. Cells were treated with 4′,6-diamidino-2-phenylindole (DAPI; 1%) for 5 min to stain nuclei, washed 3 times in 1% BSA, and mounted on slides using Prolong^®^ Gold antifade reagent. Images were collected on an Olympus Cell Vivo IX83 with a Yokogawa CSU-W1 confocal scanning unit. Z-stacks were deconvoluted in Olympus Cell Sens software using a constrained iterative algorithm. No or negligible labeling was seen in the absence of primary antibodies (data not shown). Overlays and brightness/contrast/background adjustments were carried out using ImageJ software.

### 4.12. Proximity Ligation Assay

The 8 × 10^4^ transfected PANC-1 cells were seeded on coverslips 72 h before the proximity ligation assay (PLA) experiment. As described before [61], cells were washed with PBS and fixed with PFA (4%) at room temperature for 20 min. Thereafter, cells were washed and permeabilized with 0.1% Triton^TM^ X-100 (Sigma, Saint-Quentin-Fallavier, France) for 10 min. The Duolink in situ PLA detection kit (Sigma-Aldrich, Saint-Quentin-Fallavier, France) was used to detect interactions between TRPC1 and PI3K. Experiments were performed following the manufacturer’s protocol. Red fluorescent oligonucleotides produced as the end product of the procedure were visualized using the Zeiss Observer Z1 microscope 60X oil objective (Carl Zeiss, Oberkochen, Germany). Images were analyzed using ImageJ software 1.53a (National Institute of Health, Bethesda, MD, USA), where puncta per cell were counted and normalized to the control. At least 20 pictures per condition were captured and analyzed.

### 4.13. Statistical Analysis

All data are shown as representative images or as mean measurements with standard error of means (SEM) error bars and represent at least three independent experiments. N refers to the population size, where *n* refers to the number of independent experiments performed or to the number of cells analyzed. Welch’s correction t-test or Tukey’s multiple comparison test was applied to test for statistically significant differences between two groups. *, **, ***, and **** denotes *p* < 0.05, *p* < 0.01, *p* < 0.001, and *p* < 0.0001, respectively. All graphs and statistics were generated in GraphPad Prism 9.0 software. 

## Data Availability

Not applicable.

## Supplementary Materials

The following supporting information can be downloaded at: https://www.mdpi.com/article/10.3390/ijms23147923/s1. Refs. [60,62] are cited in the supplementary materials.

## References

[1] Kleeff J., Korc M., Apte M., La Vecchia C., Johnson C.D., Biankin A.V., Neale R.E., Tempero M., Tuveson D.A., Hruban R.H. (2016). Pancreatic cancer. Nat. Rev. Dis. Primers.

[2] Siegel R.L., Miller K.D., Jemal A. (2018). Cancer statistics, 2018. CA A Cancer J. Clin..

[3] Quinonero F., Mesas C., Doello K., Cabeza L., Perazzoli G., Jimenez-Luna C., Rama A.R., Melguizo C., Prados J. (2019). The challenge of drug resistance in pancreatic ductal adenocarcinoma: A current overview. Cancer Biol. Med..

[4] Berridge M.J., Lipp P., Bootman M.D. (2000). The versatility and universality of calcium signalling. Nat. Rev. Mol. Cell Biol..

[5] Borowiec A.S., Bidaux G., Pigat N., Goffin V., Bernichtein S., Capiod T. (2014). Calcium channels, external calcium concentration and cell proliferation. Eur. J. Pharmacol..

[6] Hodeify R., Yu F., Courjaret R., Nader N., Dib M., Sun L., Adap E., Hubrack S., Machaca K., Kozak J.A., Putney J.W. (2018). Regulation and Role of Store-Operated Ca(2+) Entry in Cellular Proliferation. Calcium Entry Channels in Non-Excitable Cells.

[7] Capiod T. (2013). The need for calcium channels in cell proliferation. Recent Pat. Anticancer Drug Discov..

[8] Tajada S., Villalobos C. (2020). Calcium Permeable Channels in Cancer Hallmarks. Front. Pharmacol..

[9] Chen X., Sooch G., Demaree I.S., White F.A., Obukhov A.G. (2020). Transient Receptor Potential Canonical (TRPC) Channels: Then and Now. Cells.

[10] Wang H., Cheng X., Tian J., Xiao Y., Tian T., Xu F., Hong X., Zhu M.X. (2020). TRPC channels: Structure, function, regulation and recent advances in small molecular probes. Pharmacol. Ther..

[11] Elzamzamy O.M., Penner R., Hazlehurst L.A. (2020). The Role of TRPC1 in Modulating Cancer Progression. Cells.

[12] Faouzi M., Hague F., Geerts D., Ay A.S., Potier-Cartereau M., Ahidouch A., Ouadid-Ahidouch H. (2016). Functional cooperation between KCa3.1 and TRPC1 channels in human breast cancer: Role in cell proliferation and patient prognosis. Oncotarget.

[13] Sun Y., Ye C., Tian W., Ye W., Gao Y.Y., Feng Y.D., Zhang H.N., Ma G.Y., Wang S.J., Cao W. (2021). TRPC1 promotes the genesis and progression of colorectal cancer via activating CaM-mediated PI3K/AKT signaling axis. Oncogenesis.

[14] Zhang Y., Lun X., Guo W. (2020). Expression of TRPC1 and SBEM protein in breast cancer tissue and its relationship with clinicopathological features and prognosis of patients. Oncol. Lett..

[15] Wang A., Guo H., Long Z. (2021). Integrative Analysis of Differently Expressed Genes Reveals a 17-Gene Prognosis Signature for Endometrial Carcinoma. Biomed. Res. Int..

[16] Mandavilli S., Singh B.B., Sahmoun A.E. (2012). Serum calcium levels, TRPM7, TRPC1, microcalcifications, and breast cancer using breast imaging reporting and data system scores. Breast Cancer.

[17] Ibrahim S., Dakik H., Vandier C., Chautard R., Paintaud G., Mazurier F., Lecomte T., Gueguinou M., Raoul W. (2019). Expression Profiling of Calcium Channels and Calcium-Activated Potassium Channels in Colorectal Cancer. Cancers.

[18] Liu X., Zou J., Su J., Lu Y., Zhang J., Li L., Yin F. (2016). Downregulation of transient receptor potential cation channel, subfamily C, member 1 contributes to drug resistance and high histological grade in ovarian cancer. Int. J. Oncol..

[19] Chen L., Shan G., Ge M., Qian H., Xia Y. (2022). Transient Receptor Potential Channel 1 Potentially Serves as a Biomarker Indicating T/TNM Stages and Predicting Long-Term Prognosis in Patients with Renal Cell Carcinoma. Front. Surg..

[20] Xu Z., Shao Z., Wang M., Thorndike E., Song Y., Shang Z. (2017). Expression of transient receptor potential canonical 1 (TRPC1) in tongue squamous cell carcinoma and correlations with clinicopathological features and outcomes. Int. J. Clin. Exp. Pathol..

[21] Ke C., Long S. (2022). Dysregulated transient receptor potential channel 1 expression and its correlation with clinical features and survival profile in surgical non-small-cell lung cancer patients. J. Clin. Lab. Anal..

[22] Bollimuntha S., Singh B.B., Shavali S., Sharma S.K., Ebadi M. (2005). TRPC1-mediated inhibition of 1-methyl-4-phenylpyridinium ion neurotoxicity in human SH-SY5Y neuroblastoma cells. J. Biol. Chem..

[23] Bomben V.C., Sontheimer H. (2010). Disruption of transient receptor potential canonical channel 1 causes incomplete cytokinesis and slows the growth of human malignant gliomas. Glia.

[24] Asghar M.Y., Magnusson M., Kemppainen K., Sukumaran P., Lof C., Pulli I., Kalhori V., Tornquist K. (2015). Transient Receptor Potential Canonical 1 (TRPC1) Channels as Regulators of Sphingolipid and VEGF Receptor Expression: Implications for Thyroid Cancer Cell Migration and Proliferation. J. Biol. Chem..

[25] Kaemmerer E., Turner D., Peters A.A., Roberts-Thomson S.J., Monteith G.R. (2018). An automated epifluorescence microscopy imaging assay for the identification of phospho-AKT level modulators in breast cancer cells. J. Pharmacol. Toxicol. Methods.

[26] El Hiani Y., Ahidouch A., Lehen’kyi V., Hague F., Gouilleux F., Mentaverri R., Kamel S., Lassoued K., Brule G., Ouadid-Ahidouch H. (2009). Extracellular signal-regulated kinases 1 and 2 and TRPC1 channels are required for calcium-sensing receptor-stimulated MCF-7 breast cancer cell proliferation. Cell Physiol. Biochem..

[27] El Hiani Y., Lehen’kyi V., Ouadid-Ahidouch H., Ahidouch A. (2009). Activation of the calcium-sensing receptor by high calcium induced breast cancer cell proliferation and TRPC1 cation channel over-expression potentially through EGFR pathways. Arch. Biochem. Biophys..

[28] Tajeddine N., Gailly P. (2012). TRPC1 protein channel is major regulator of epidermal growth factor receptor signaling. J. Biol. Chem..

[29] Selli C., Erac Y., Kosova B., Erdal E.S., Tosun M. (2015). Silencing of TRPC1 regulates store-operated calcium entry and proliferation in Huh7 hepatocellular carcinoma cells. Biomed. Pharmacother..

[30] Selli C., Pearce D.A., Sims A.H., Tosun M. (2016). Differential expression of store-operated calcium- and proliferation-related genes in hepatocellular carcinoma cells following TRPC1 ion channel silencing. Mol. Cell Biochem..

[31] Zeng B., Yuan C., Yang X., Atkin S.L., Xu S.Z. (2013). TRPC channels and their splice variants are essential for promoting human ovarian cancer cell proliferation and tumorigenesis. Curr. Cancer Drug Targets.

[32] Zeng Y.Z., Zhang Y.Q., Chen J.Y., Zhang L.Y., Gao W.L., Lin X.Q., Huang S.M., Zhang F., Wei X.L. (2021). TRPC1 Inhibits Cell Proliferation/Invasion and Is Predictive of a Better Prognosis of Esophageal Squamous Cell Carcinoma. Front. Oncol..

[33] Zhang L.Y., Zhang Y.Q., Zeng Y.Z., Zhu J.L., Chen H., Wei X.L., Liu L.J. (2020). TRPC1 inhibits the proliferation and migration of estrogen receptor-positive Breast cancer and gives a better prognosis by inhibiting the PI3K/AKT pathway. Breast Cancer Res. Treat.

[34] Selli C., Erac Y., Tosun M. (2015). Simultaneous measurement of cytosolic and mitochondrial calcium levels: Observations in TRPC1-silenced hepatocellular carcinoma cells. J. Pharmacol. Toxicol. Methods.

[35] Madsen C.P., Klausen T.K., Fabian A., Hansen B.J., Pedersen S.F., Hoffmann E.K. (2012). On the role of TRPC1 in control of Ca^2+^ influx, cell volume, and cell cycle. Am. J. Physiol. Cell Physiol..

[36] Davis F.M., Peters A.A., Grice D.M., Cabot P.J., Parat M.O., Roberts-Thomson S.J., Monteith G.R. (2012). Non-stimulated, agonist-stimulated and store-operated Ca^2+^ influx in MDA-MB-468 breast cancer cells and the effect of EGF-induced EMT on calcium entry. PLoS ONE.

[37] El Boustany C., Bidaux G., Enfissi A., Delcourt P., Prevarskaya N., Capiod T. (2008). Capacitative calcium entry and transient receptor potential canonical 6 expression control human hepatoma cell proliferation. Hepatology.

[38] Lepannetier S., Zanou N., Yerna X., Emeriau N., Dufour I., Masquelier J., Muccioli G., Tajeddine N., Gailly P. (2016). Sphingosine-1-phosphate-activated TRPC1 channel controls chemotaxis of glioblastoma cells. Cell Calcium.

[39] Rychkov G., Barritt G.J. (2007). TRPC1 Ca(2+)-permeable channels in animal cells. Handb. Exp. Pharmacol..

[40] Hong J.H., Li Q., Kim M.S., Shin D.M., Feske S., Birnbaumer L., Cheng K.T., Ambudkar I.S., Muallem S. (2011). Polarized but differential localization and recruitment of STIM1, Orai1 and TRPC channels in secretory cells. Traffic.

[41] Kim M.H., Seo J.B., Burnett L.A., Hille B., Koh D.S. (2013). Characterization of store-operated Ca^2+^ channels in pancreatic duct epithelia. Cell Calcium.

[42] Dong H., Shim K.N., Li J.M., Estrema C., Ornelas T.A., Nguyen F., Liu S., Ramamoorthy S.L., Ho S., Carethers J.M. (2010). Molecular mechanisms underlying Ca^2+^-mediated motility of human pancreatic duct cells. Am. J. Physiol. Cell Physiol..

[43] Ravi M., Paramesh V., Kaviya S.R., Anuradha E., Solomon F.D. (2015). 3D cell culture systems: Advantages and applications. J. Cell Physiol..

[44] Ambudkar I.S., de Souza L.B., Ong H.L. (2017). TRPC1, Orai1, and STIM1 in SOCE: Friends in tight spaces. Cell Calcium.

[45] Sobradillo D., Hernandez-Morales M., Ubierna D., Moyer M.P., Nunez L., Villalobos C. (2014). A reciprocal shift in transient receptor potential channel 1 (TRPC1) and stromal interaction molecule 2 (STIM2) contributes to Ca^2+^ remodeling and cancer hallmarks in colorectal carcinoma cells. J. Biol. Chem..

[46] Saez-Rodriguez J., MacNamara A., Cook S. (2015). Modeling Signaling Networks to Advance New Cancer Therapies. Annu. Rev. Biomed. Eng..

[47] Azimi I., Milevskiy M.J.G., Kaemmerer E., Turner D., Yapa K., Brown M.A., Thompson E.W., Roberts-Thomson S.J., Monteith G.R. (2017). TRPC1 is a differential regulator of hypoxia-mediated events and Akt signalling in PTEN-deficient breast cancer cells. J. Cell Sci..

[48] Fabian A., Bertrand J., Lindemann O., Pap T., Schwab A. (2012). Transient receptor potential canonical channel 1 impacts on mechanosignaling during cell migration. Pflug. Arch..

[49] Fels B., Bulk E., Petho Z., Schwab A. (2018). The Role of TRP Channels in the Metastatic Cascade. Pharmaceuticals.

[50] Park W., Chawla A., O’Reilly E.M. (2021). Pancreatic Cancer: A Review. JAMA.

[51] Perrouin-Verbe M.A., Schoentgen N., Talagas M., Garlantezec R., Uguen A., Doucet L., Rosec S., Marcorelles P., Potier-Cartereau M., Vandier C. (2019). Overexpression of certain transient receptor potential and Orai channels in prostate cancer is associated with decreased risk of systemic recurrence after radical prostatectomy. Prostate.

[52] Perrouin Verbe M.A., Bruyere F., Rozet F., Vandier C., Fromont G. (2016). Expression of store-operated channel components in prostate cancer: The prognostic paradox. Hum. Pathol..

[53] Bonelli P., Tuccillo F.M., Borrelli A., Schiattarella A., Buonaguro F.M. (2014). CDK/CCN and CDKI alterations for cancer prognosis and therapeutic predictivity. Biomed. Res. Int..

[54] De Luca A., Maiello M.R., D’Alessio A., Pergameno M., Normanno N. (2012). The RAS/RAF/MEK/ERK and the PI3K/AKT signalling pathways: Role in cancer pathogenesis and implications for therapeutic approaches. Expert Opin. Ther. Targets.

[55] Roy S.K., Srivastava R.K., Shankar S. (2010). Inhibition of PI3K/AKT and MAPK/ERK pathways causes activation of FOXO transcription factor, leading to cell cycle arrest and apoptosis in pancreatic cancer. J. Mol. Signal.

[56] Nussinov R., Wang G., Tsai C.J., Jang H., Lu S., Banerjee A., Zhang J., Gaponenko V. (2017). Calmodulin and PI3K Signaling in KRAS Cancers. Trends Cancer.

[57] Zanou N., Schakman O., Louis P., Ruegg U.T., Dietrich A., Birnbaumer L., Gailly P. (2012). Trpc1 ion channel modulates phosphatidylinositol 3-kinase/Akt pathway during myoblast differentiation and muscle regeneration. J. Biol. Chem..

[58] Chaudhuri P., Rosenbaum M.A., Sinharoy P., Damron D.S., Birnbaumer L., Graham L.M. (2016). Membrane translocation of TRPC6 channels and endothelial migration are regulated by calmodulin and PI3 kinase activation. Proc. Natl. Acad. Sci. USA.

[59] Jonckheere N., Van Seuningen I. (2018). Integrative analysis of the cancer genome atlas and cancer cell lines encyclopedia large-scale genomic databases: MUC4/MUC16/MUC20 signature is associated with poor survival in human carcinomas. J. Transl. Med..

[60] Radoslavova S., Folcher A., Lefebvre T., Kondratska K., Guenin S., Dhennin-Duthille I., Gautier M., Prevarskaya N., Ouadid-Ahidouch H. (2021). Orai1 Channel Regulates Human-Activated Pancreatic Stellate Cell Proliferation and TGFbeta1 Secretion through the AKT Signaling Pathway. Cancers.

[61] Chamlali M., Kouba S., Rodat-Despoix L., Todesca L.M., Petho Z., Schwab A., Ouadid-Ahidouch H. (2021). Orai3 Calcium Channel Regulates Breast Cancer Cell Migration through Calcium-Dependent and -Independent Mechanisms. Cells.

[62] Pfaffl M.W., Horgan G.W., Dempfle L. (2002). Relative expression software tool (REST) for group-wise comparison and statistical analysis of relative expression results in real-time PCR. Nucleic Acids Res..

