# Supplementary material for: The TRPC1 Channel Forms a PI3K/CaM Complex and Regulates Pancreatic Ductal Adenocarcinoma Cell Proliferation in a Ca2+-Independent Manner
Source: Int J Mol Sci. 2022 Jul 18;23(14):7923. doi: 10.3390/ijms23147923 (PMC9323718; doi:10.3390/ijms23147923)
Supplement: Supplementary file 1 [file ijms-23-07923-s001.zip › ijms-1814953-supplementary.pdf]

International Journal of
Molecular Sciences

MDPI

Supplementary Figures

The TRPC1 channel forms a PI3K/CaM complex and regulates pancreatic ductal adenocarcinoma cell proliferation in a Ca^{2+} -independent manner

Julie Schnipper[1], Sana Kouba[1], Frédéric Hague[1], Alban Girault[1], Pierre Rybarczyk[1,2], Marie-Sophie Telliez[1], Stéphanie Guénin[3], Riad Tebbakha[1,2], Henri Sevestre[1,2], Ahmed Ahidouch[1,4], Stine Falsig Pedersen[5], Halima Ouadid-Ahidouch[1]*

[1] Laboratory of Cellular and Molecular Physiology, UR UPJV 4667, University of Picardie Jules Verne, Amiens, France; julie_schnipper@hotmail.com (JS), Sana Kouba sana.kouba@hotmail.com (SK), fh-lnc@u-picardie.fr (FH), alban.girault@u-picardie.fr (AG), marie-sophie.telliez@u-picardie.fr (MST),

[2] Anatomy and Pathology Department, Amiens Hospital, University of Picardie Jules Verne, Tumorotheque of Picardie, Amiens, France; Rybarczyk.Pierre@chu-amiens.fr (PR), Henrisevestre@gmail.com (HS), Tebbakha.Riad@chu-amiens.fr (RT)

[3] Centre de Ressources Régional en Biologie Moléculaire, Université de Picardie Jules Verne, Amiens, France; stephanie.vandecasteele@u-picardie.fr (SG)

[4] Biology department, University Ibn Zohr, Agadir, Morocco, ahidouch@gmail.com (AA)

[5] Section for Cell Biology and Physiology, Department of Biology, University of Copenhagen, Copenhagen Ø, Denmark; sfpedersen@bio.ku.dk (SFP)

* Correspondence: halima.ahidouch-ouadid@u-picardie.fr (HOA); Tel.: +33-322827646

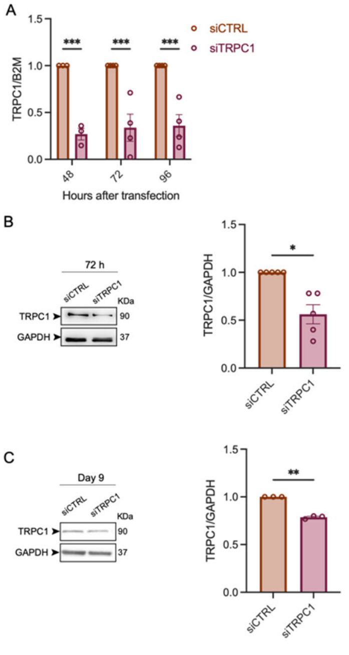

Figure S1: **(A)** TRPC1 mRNA expression evaluated by qPCR 48, 72 and 96 hours after transfection with siCTRL and siTRPC1 in PANC-1 lines (n = 3-4). Materials and methods for qPCR are described below. **(B)** TRPC1 protein expression evaluated 72 hours after transfection with siCTRL and siTRPC1 in PANC-1 lines (n = 5). **(B)** TRPC1 protein expression evaluated 9 days after transfection with siCTRL and siTRPC1 in PANC-1 spheroids (n = 3). *, ** and *** indicate p < 0.05, 0.01 and 0.001, respectively.

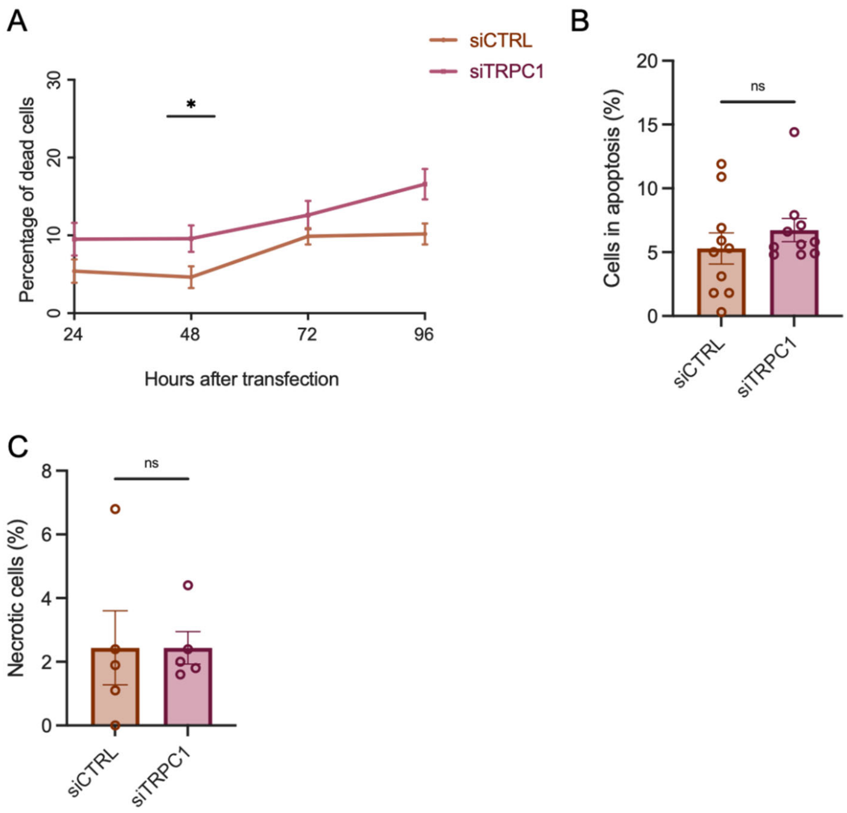

Figure S2: (A) Percentage of dead cells analysed by the Trypan Blue Assay after 24, 48, 72 and 96 h after transfection with siCTRL and siTRPC1 in PANC-1 cells, respectively (n = 4). **(B)** Quantification of Annexin-5 analysis of cells in apoptosis 72 h after transfection with siCTRL and siTRPC1 in PANC-1 cells (n = 3). Methods and materials for Annexin-5 analysis are described below. **(C)** Quantification of Annexin-5 analysis of cells necrotic cells 72 h after transfection with siCTRL and siTRPC1 in PANC-1 cells (n = 3). Methods and materials for Annexin-5 analysis are described below.). * indicate $p < 0.05$ and ns indicate non-significant.

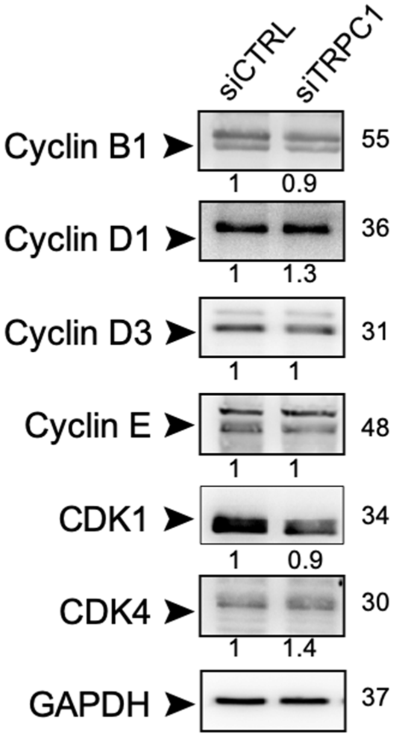

Figure S3: Westernblot analysis of different cell cycle regulating proteins 72 h after transfection with siCTRL and siTRPC1 in PANC-1 cells (n= 3-4, no significance was found).

Supplemental Materials and Methods

Quantitative Real-Time PCR (qRT-PCR)

Total RNA was extracted with the Trizol reagent (Sigma, Saint-Quentin-Fallavier, France) method as previously described [60]. RNA concentration and purity were determined using a spectrophotometer (NanoDrop 2000, Wilmington, NC, USA). 2 µg of RNA was converted into cDNA with the MultiScribe™ Reverse Transcriptase kit (Applied Biosystems, Carlsbad, CA, USA). Real-time PCR was performed on a LightCycler 480 System (Roche, Basel, Switzerland) using SYBR Green I PCR master mix (Life Science, Roche, Basel, Switzerland), and sense and antisense PCR primers specific to TRPC1 (forward 5′GAGGTGATGGCGCTGAAGG-3′ and reverse 5′-GCACGCCAGCAAGAAAAGC-3′) and B2M (forward 5′GTCTTTCAGCAAGGACTGGTC3′ and reverse CAAATGCGGCATCTTCAAACC3′). TRPC1 mRNA expression was normalized to B2M, used as housekeeping gene, and compared to the control sample, using the Pfaffl method [62].

FACS analysis of annexin V and propidium iodide staining

To evaluate the percentage of apoptotic and necrotic cells, we detected phosphatidylserine residues at the outer plasma membrane by the FITC Annexin V Apoptosis Detection Kit I (BD Biosciences Pharmingen, Le Pont-de-Claix, France). 5×10^5 transfected PANC-1 cells were seeded, and collected after 72 h. Both detached and adherent cells were collected by trypsinization. Then the cells were pelleted, washed twice with ice-cold PBS and resuspended in 1× binding buffer (BD Biosciences

Pharmingen). Following the PE Annexin V Apoptosis Detection Kit staining protocol, we added FITC Annexin V and propidium iodide to the cell pellets and incubated them at 25°C in the dark for 15 min. Binding buffer was then added to each tube and samples were directly analysed by the flow cytometer (Accuri®) in order to determine the percentage of apoptotic cells.

References

60. Radoslavova, S.; Folcher, A.; Lefebvre, T.; Kondratska, K.; Guenin, S.; Dhennin-Duthille, I.; Gautier, M.; Prevarskaya, N.; Ouadid-Ahidouch, H., Orai1 Channel Regulates Human-Activated Pancreatic Stellate Cell Proliferation and TGFbeta1 Secretion through the AKT Signaling Pathway. *Cancers (Basel)* **2021,** 13, (10), DOI: 10.3390/cancers13102395.

62. Pfaffl, M. W.; Horgan, G. W.; Dempfle, L., Relative expression software tool (REST) for group-wise comparison and statistical analysis of relative expression results in real-time PCR. *Nucleic Acids Res* **2002,** 30, (9), e36, DOI: 10.1093/nar/30.9.e36.
